# The potentials of digital technology in social prescribing: a qualitative study of key stakeholders’ perspectives

**DOI:** 10.1186/s12889-025-23091-7

**Published:** 2025-05-31

**Authors:** Sima Rafiei, Mahsa Honary, Barbara Mezes

**Affiliations:** 1 Dept. Management Science, Lancaster University Management School, Lancaster, UK; 2 Department of Primary Care and Mental Health, University of Liverpool, Liverpool, UK

**Keywords:** Information technology, Digitalisation, Social prescribing, Advantages, Challenges

## Abstract

**Background:**

The automation and effective communication provided by technological facilities allow service providers to deliver social wellbeing activities in a prompt and cost-effective manner. This study explored the perspectives of social prescribers, coordinators, and community providers on the potential of digital technology in social prescribing and the challenges to its implementation.

**Methods:**

We conducted a qualitative study and held three Focus Group Discussion (FGD) sessions with a sample of 18 stakeholders in the Northwest of England, UK including social prescribing coordinators, social prescribers, and voluntary, community, faith, and social enterprise (VCFSE) sector providers^1^ to qualitatively explore the responses of different participant groups and reveal a wealth of deep insight around the study objective. A purposive sampling strategy was used to select information-rich participants, providing in-depth insights. Transcriptions were analysed through thematic analysis supported by Max QDA to identify common themes and ideas that were generated across the focus groups.

**Results:**

Eight themes were identified regarding the potentials and challenges of digital social prescribing from the study participants’ viewpoints. The main themes highlighting advantages include capacity and resource management, coordination and integration of support services, access and equity, and outcome evaluation. The challenges identified are digital illiteracy, awareness concerns, financial matters, and a lack of evidence.

**Conclusions:**

Our findings confirm that digital social prescribing offers significant potential to improve accessibility, time efficiency, and personalization in service delivery. However, they also underscore critical challenges, including technical barriers, financial constraints, and a lack of digital literacy among users and providers. Stakeholders emphasized that while digital SP can streamline referrals and enhance data sharing, its success depends on addressing inequalities in access to technology.

## Background

Worldwide, health and social care systems face increasing demand due to population ageing, rising chronic disease prevalence, and the emergence of advanced diagnostic and treatment technologies. Many services—including the National Health Service (NHS)—struggle with insufficient sustainable funding to meet the population’s complex needs [1, 2]. Historically, healthcare efforts and resources focused mainly on biomedical and clinical approaches since the birth of social medicine in France and Germany in 1848; the medical model has remained dominant ever since [3]. To broaden this focus, healthcare systems globally have promoted social prescribing—linking health professionals with community services—to improve mental and social wellbeing [3, 4]. Social prescribing emphasizes social determinants of health (SDH) and connects individuals with nonmedical support within their communities [4, 5].

As a holistic approach, social prescribing allows professionals to refer individuals to communitybased services that address social, emotional, and practical needs impacting health. Factors such as social isolation, housing conditions, and financial hardship significantly affect outcomes. By connecting people to resources like social clubs, exercise groups, or financial advice services, social prescribing aims to enhance quality of life and reduce pressure on traditional healthcare systems [6]. Moreover, social prescribing not only eases the burden on clinical services but also lowers perperson costs compared with traditional public health interventions [7]. In 2021/22, the Department of Health and Social Care reported that most of the NHS budget funded GP surgeries, mental health provisions, and hospital services, while local authorities allocated a smaller share to social care initiatives [5]. The US Centers for Disease Control and Prevention (CDC) highlights that strong social connections protect against serious illness: individuals with robust social bonds live longer, experience less anxiety, and enjoy better physical health [8].

Studies have shown that digital social prescribing (DSP) frees link workers and service providers to focus on those with the greatest need [9]. Integrating digital tools into social prescribing more effectively addresses social determinants, improves health outcomes, and promotes efficient resource use [9]. Currently, several digital social prescription tools (DSPTs) are in use in the UK for people with chronic physical conditions. These systems leverage electronic patient records and online directories to prescribe personcentred nonmedical services to those who struggle to leave home [10, 11]. This remote capability enables link workers and GPs to consult with individuals over long distances. Technological advances and new devices also enhance service quality by analysing diverse patient information—medical history, medications, clinical symptoms, lifestyle factors, and social determinants—to tailor personalised care plans [12, 13].

Digital technology should streamline the collection of data on service users, providers, and referral pathways, while enabling flexible, ongoing monitoring to ensure continuous support [10]. Several studies call for technologyenabled social prescribing platforms that facilitate referrals, aggregate client health data, and include builtin wellbeing measures to rapidly identify emerging needs [14–16]. Future applications may involve digital signposting to appropriate services and automated reminders to prompt timely access to care [17–19]. Despite its promise, DSP faces challenges—particularly for smaller social prescribing services—that could hinder widespread adoption.

### Implications of the study

Despite the fact that a few numbers of studies have been conducted to explore the opportunities and challenges around the use of digital technology in the SP system, there is still an important necessity to discover broad applications of this technology from the viewpoints of different health and social care professionals including link workers, health and wellbeing coordinators, and community providers. Furthermore, identifying gaps in digital service delivery and challenges that interfere with the adoption and implementation of this approach on a large scale are among key research questions that require a solicitation response.

### Objective

This study aimed to increase our understanding about the viewpoints of three different stakeholders’ groups including social prescribers, community providers and social prescribing coordinators on the potentials of digital technology in social prescribing and existing challenges towards the realisation of its objectives.

## Methods

We employed a qualitative research design, conducting three focus groups to gather in-depth insights from stakeholders involved in social prescribing. The number of focus groups was determined based on achieving data saturation and ensuring a diverse range of perspectives. We chose the focus group method for its ability to facilitate productive interactions and open dialogue among participants. The initial phase involved the creation of discussion topic guide, which was informed by insights gathered through a comprehensive literature review and expert input. This guide centred around exploring the potential advantages and challenges associated with integrating digital technology into social prescribing. It also aimed to assess the feasibility of incorporating a digital platform into future social prescribing care infrastructure, with the goal of enhancing both the utilisation of social prescribing services and the overall quality of care. The guide covered topics related to the use of information technology and digital resources during the system’s implementation, as well as an examination of the facilitators and barriers that service providers might encounter when considering the integration of digital tools.

### Study design

To collect an all-inclusive information about the stakeholders’ viewpoint toward the opportunities and challenges that might arise from digital technology in social prescribing, qualitative research study was conducted across the Lancashire and South Cumbria area, United Kingdom.

### Study participants

We conducted three focusgroup discussions (FGDs) with 18 stakeholders in Northwest England, UK: (i) NHS service commissioners/providers, (ii) link workers, and (iii) communitybased social prescribing providers. Each homogeneous group comprised 5–7 participants. Ethical approval was granted by Lancaster University and the Health Research Authority. Eligible participants had experience with social prescribing in Lancashire and South Cumbria, were aged ≥ 18 years, and could give informed consent.

### Recruitment

Participants were identified via project partners and the Northwest Clinical Research Network (CRN). NHS commissioners from the Healthier Lancashire and South Cumbria ICS, link workers, and community providers meeting our inclusion criteria were invited by email or telephone. Additional stakeholders were recruited via snowball sampling.

### Procedure

Inperson FGDs took place between August and September 2023 at community venues and Lancaster University. Each 2.5–3 h session (including a short break) was moderated by a researcher using a semistructured topic guide. After a brief introduction to the study aims, participants discussed their experiences with social prescribing and explored how digital tools might improve referral processes and wellbeing measurement. Creative, activityoriented exercises (e.g. mapping current workflows, envisioning digital enhancements) encouraged engagement. All sessions were audiorecorded, and participants completed a brief demographic questionnaire.

### Data analysis

Data were analysed using inductive thematic analysis in MaxQDA, following Braun and Clarke’s (2021) guidelines [20]. One researcher reviewed all transcripts to identify meaningful statements, applied descriptive codes, and then organized these codes into themes—paying particular attention to illustrative participant quotations. Finally, the findings were organized into major themes, categories, and supporting quotations.

## Findings

### Characteristics of study participants

Table 1 depicts the characteristics of study participants.

**Table 1:** Study participants’ characteristics (*N* = 18)

Characteristics		Count (*n*)	Distribution (%)
Gender	Male	4	22.2
	Female	14	77.8
Age	30–40	8	44.4
	40–50	6	33.3
	≥ 50	4	22.3
Job title	Integrated care community development lead	1	5.5
	Head of public protection	1	5.5
	Healthier Lancashire and south Cumbria ICS lead	1	5.5
	Community health officer	1	5.5
	Service Development Manager	1	5.5
	Community VCFSE sector provider	7	38.8
	Social prescriber	6	33.7
Length of service	< 10	6	33.3
	10–20	8	44.4
	≥ 20	4	22.3

### Themes developed from the analysis

As shown in Table 2, we identified eight main themes—four reflecting the advantages of digital social prescribing and four outlining its challenges, each with 2–4 subthemes.

**Table 2:** Themes and Sub-themes of respondents’ viewpoint toward digital social prescribing potentials and challenges

Categories	Potentials	
	Main themes	Sub-themes
Advantages	Capacity and resource management	• Workload implications • Time efficiency and service effectiveness
	Coordination and integration of support services	• Integration with existing systems • Inter-provider communication and continuity of care • Referral processes and information sharing
	Access and equity	• Accessibility to community well-being activities • The provision of tailored-based services • Self-reliance
	Outcome evaluation	• Digital SP as a tool to monitor key performance indicators (KPIs) • Continuous evaluation of the referral programme • Assessing the effectiveness of community-based support services
Challenges	Digital illiteracy	• Technical difficulties • Data insecurity
	Awareness concerns	• Lack of awareness • Lack of skills
	Financial matters	• Financial insecurity and lack of infrastructural resources
	Lack of evidence	• Lack of evidence on the effectiveness of digital SP • Stakeholder scepticism

### Advantages

#### Capacity and resource management

##### Workload implications

This sub-theme corresponds to how digital social prescribing tools can alleviate or add to the workload of healthcare providers. Most service providers agreed that integrating digitalisation into social prescribing allows them additional time to engage with service users more effectively. They emphasised that establishing strong relationships with service users requires dedicated time for fostering friendly and empathetic interactions. Several participants highlighted that a successful social prescribing programme needs adequate time and resources especially when dealing with service users with complicated mental health or social needs. Several link workers added that having sufficient time enables them to build a deeper understanding of service users, fostering an atmosphere of mutual understanding and commitment to address their unmet needs and concerns. *“Most of the time I’m busy with doing admin works rather than spending time with users. We need time to develop trust and closer relationship with people*,* so they feel free to discuss their concerns openly”* (SP2). This sentiment was echoed by others who emphasised that digital tools, by streamlining tasks, allow staff to better meet complex social and mental health needs.

##### Time efficiency and service effectiveness

Participants agreed that digital SP systems could increase efficiency by reducing manual tasks and enabling timely service delivery. SP8 stated, “It’s really important to design technology-based SP in a way that it delivers more flexible interventions to people in a timelier manner and more reasonable cost.” Access to real-time data was also seen as a key enabler for personalised care, as SP4 described: “Literally, using digital social prescribing provides an opportunity for service providers to share their best experiences electronically, and improves the flow of people in the social prescribing system.”

#### Coordination and integration of support services

##### Integration with existing systems

Several participants noted the importance of integrating DSP with existing NHS and local authority systems. SP12 envisioned, “A group of digital technicians can simply develop and implement an integrated electronic social prescribing referral system with electronic patient records.” SP2 added that such integration improves community connectedness: “Digital social prescribing lets patients feel more connected to their community.” SP7 emphasized the value of secure systems to ensure privacy: “This shared vision will help the whole health system develop participatory plans for digitally person-based services… with maximum information security.”

##### Inter-provider communication and continuity of care

Participants recognised that digital SP platforms enhance continuity by allowing real-time tracking and uninterrupted service delivery. SP7 highlighted, “I always feel a gap in the social prescribing system which mainly deals with inconsistent tracking… and delivering real-time feedback.” SP10 added that digitisation can offload certain burdens: “Sometimes you think you are overwhelmed with several tasks… which can be easily removed by digitalisation.”

##### Referral processes and information sharing

Improved information sharing and streamlined referrals were recurring themes. SP5 commented, “E-referrals can avoid pointless referrals; they can provide effective care to the right people in the most proper setting and time.” SP11 saw efficiency gains: “Using technology can minimise the need for paperwork and manual requests for users’ information.” SP15 emphasized reducing disparities through better system integration: “Digital technologies can reduce disparities in health outcomes if we simplify the process of technology… so that data flows effortlessly.” Participants also noted how DSP enhances staff learning and skill-building. SP18 suggested: “Why not try online courses for social support counselling methods?”

Participants also believed that integrating community directory software with electronic patient records could facilitate streamlined referrals to non-therapeutic activities that precisely match their social needs. They further noted that this approach enables patient-centred interventions in non-clinical settings, enhancing the overall effectiveness of interventions. *“Don’t you think digital technologies can reduce disparities in health outcomes if we simplify the process of technology to users to interact with the system in a way that makes data flow effortlessly between different information systems?” (SP15).*

#### Access and equity

##### Accessibility to community well-being activities

Digital SP was seen as essential for improving access, especially for people in rural or underserved areas. SP13 observed, “Online consultation services… let users access services anywhere, anytime based on what they actually need.” Participants added that online platforms help manage workload and increase care continuity.

##### Tailored-based services

Many participants agreed that a digital social prescribing system aims to provide personalised and tailored services aligned with users’ needs and expectations. They explained that designing the system in such a way that it matches activities to individuals’ preferences, comorbidities, and locality would tailor non-medical interventions to address their needs efficiently. They underscored that social prescribing services should complement clinical approaches by providing support targeting the social determinants of health and non-medical factors impacting their wellbeing. Another fundamental requirement mentioned by study participants was the design of an information system facilitating connections across various healthcare settings, while providing essential information about clients’ health conditions, socio-economic status, and medical backgrounds. *“Even clinicians and GPs should be informed of the key role of social factors like housing*,* education*,* income*,* and a wider range of environmental factors” (SP9).*

##### Self-reliance

Some service providers expressed that fostering digital skills and knowledge among individuals not only instils self-confidence in addressing their social needs but also empowers them to self-monitor and self-manage their health and wellbeing. According to the interviewees, involving service users in the design of the digital platform is a practical approach to ensure system compliance with users’ needs. This strategy also enhances their social engagement, boosts their self-confidence, and ensures the acceptance of the system among a larger number of clients. “*Sure*,* when we assign a piece of work or responsibility to people*,* umm*,* I mean when the responsibility is shared among different community members and then step-by-step*,* we try to enable them believe on themselves*,* be self-reliant and depend on their own capabilities” (SP5).*

#### Outcome evaluation

##### Digital SP as a tool to monitor Key Performance Indicators (KPIs)

Among various capabilities that can be mentioned for digital social prescribing, some of the study participants acknowledged it as a helping hand in providing key system performance indicators that enable them measure users’ experience and their health outcomes. *“Why shouldn’t we think of a digital platform empowering us to better measure the impact of community services on people’s health and wellbeing?” (SP1).* Some of the study participants emphasised the necessity of developing a digital social care record capable of documenting all information related to the social care services provided to individuals throughout the social prescribing pathway. “*If we see digital social prescribing within a broader context*,* more potentials will move forward” (SP4).*

##### Continuous evaluation of the referral programme

They believed that replacing paper records by a digital system would provide up-to-date electronic records that contain individual’s health and social care information. When questioned about digital systems, some of the participants stressed the need for more knowledge regarding the prospect of digitally recording social care services for service users. Others advocated for the digital recording system to enable continuous evaluation of the referral programme through a user-friendly application. *“The data sharing feature of digitalisation enables us get easy access to some of the relevant clinical history of users*,* their socio-economic factors*,* their life-style manner and any other evidence that help us in making best decisions for users” (SP4).*

##### Assessing the effectiveness of Community-based support services

There was strong support for DSP as a tool to assess and improve community-based care. SP13 stated, “It would be a fabulous opportunity to assess our progress and reinforce our accountability.” SP6 noted the value of real-time updates: “The system can be developed in a way that… GPs can be informed of the quality of community care services provided.” SP13 also highlighted the non-clinical impact: “I feel passionate about seeing people having an increased level of quality of life without receiving any medication or clinical care.”

### Challenges

#### Digital illiteracy

##### Technical difficulties

Participants identified varying levels of digital competence as a barrier to engaging with SP tools. Service providers acknowledged the need for both users and staff to acquire technical skills to effectively navigate digital systems. SP14 warned, “Not only raising awareness of digital technologies across the whole ICS and end-users is key, but also the ability to create connection between different record systems is a major risk which might encounter several errors in the functionality of the system.”

##### Data insecurity

Some of the participants also felt concern of some data security issues or complex processes that might come out of digitalisation. *“Without training it would be a big challenge”* (SP18).

##### Awareness concerns

The potential for digital SP systems to either bridge or widen gaps in service accessibility was another key concern mentioned by some of the study participants.

##### Lack of awareness

Many participants admitted to limited knowledge of existing DSP platforms and how they function. SP16 commented, “If anything really exists, we should know what it involves, or what advantages it has. Lack of information about this critical subject will lead to failure for sure.” To address this gap, some suggested more visibility and targeted communication. SP8 added, “The fundamental factor for the success of digital social prescribing is giving different people with various backgrounds adequate knowledge they need to successfully work with the system.”

##### Lack of skills

Users’ empowerment and skill building were two important strategies mentioned by service users to facilitate the development of ongoing technologies in SP area. *“What do we call it? Yeah! skill building is essential as it ensures that everyone has access to the knowledge” (SP9).*

#### Financial matters

##### Financial insecurity and lack of infrastructural resources

Almost all participants pointed to funding constraints as a major hurdle for implementing DSP. The costs of developing and maintaining digital infrastructure were frequently cited. SP15 asked, “Do we afford for paying expensive services and procurement support for digital investments?” Others emphasized the often-overlooked importance of financial guidance. SP11 noted, “It might act as a safety valve; most of the time we forget about financial and logistic services which are of great importance.”

#### Lack of evidence

##### Lack of data on the effectiveness of digital SP

One of the challenges that has been mentioned by a few study participants was lack of evidence around the impact and effectiveness of digital social prescribing. Participants highlighted a scarcity of robust studies demonstrating the impact and effectiveness of digital social prescribing (SP) interventions. This absence of empirical data leads to scepticism among stakeholders regarding the efficacy of digital SP solutions. They added that lack of longitudinal studies aimed at evaluating the impact of digitalisation on social health needs, mental health conditions, or health-related quality of life is a significant challenge that prevents different stakeholders from believing in the effectiveness of these technologies. There is a recognized gap in long-term studies assessing the effects of digitalization on social health needs, mental health conditions, and health-related quality of life. *“To my knowledge*,* there are inadequate number of studies agreeing on the outcomes of digital social prescribing and there are still lots of unknowns” (SP17).*

##### Stakeholder scepticism

This lack of data contributed to scepticism among some stakeholders, which could inhibit wider adoption. SP14 reflected, “Don’t we need some more basic information about the effectiveness of such approaches and even their cost saving effects?” Others agreed that visible and accessible evidence would help secure greater buy-in from healthcare professionals and policy leaders.

These challenges highlight the need for systemic investment not just in infrastructure, but also in skills, evidence generation, and communication strategies to support equitable and sustainable implementation of digital SP.

## Discussion

Our findings confirm that digital social prescribing (SP) offers considerable potential to improve accessibility, efficiency, and personalisation in the delivery of health and social care. Participants consistently emphasised the ability of digital tools to streamline referrals, enhance time management, reduce provider burden, and support more tailored services. However, successful implementation is contingent upon addressing multiple challenges including technical difficulties, digital exclusion, funding limitations, and system integration barriers.

Digital health technologies have become increasingly embedded in healthcare systems worldwide. With the rise in mobile apps, online platforms, remote monitoring tools, and wearable devices, the use of information technology to promote better population health and wellbeing has grown exponentially [21–23]. These technologies, whether complex (such as AI or genomics) or common (such as electronic patient records and digital care apps), are designed to optimise service delivery, enhance communication, and support patients in managing their health [24, 25]. In our study, stakeholders echoed this perspective, noting that digital SP improves information sharing, saves time, supports continuity of care, and fosters personalised support. These insights align with national policies advocating for the wider use of digital tools to improve public health outcomes and system efficiency [26, 27].

The timesaving and workload-reducing benefits of digital SP were particularly emphasised by participants. Digitalisation was viewed as enabling more meaningful engagement with service users, especially those with complex needs. These findings align with earlier studies noting the potential for digital SP to free up providers’ time and facilitate more responsive, flexible, and user-centred care [9, 10]. Additionally, participants viewed digital platforms as a way to improve access for users facing transportation, mobility, or geographic barriers—reflecting literature that identifies telehealth and online services as tools to overcome systemic access issues [28].

Several studies have also highlighted the cost-saving aspects of digital SP, particularly in reducing unnecessary referrals, minimising commuting expenses for users, and improving service allocation efficiency [10, 29]. Our study adds to this evidence by showing how digital SP enhances capacity and system responsiveness through data integration and improved referral coordination. The benefits extended beyond efficiency; participants also linked digital SP to improved self-reliance among service users, especially when platforms were co-designed and user-friendly. These findings echo previous research highlighting digital SP’s potential to support self-management, especially among people with long-term conditions [15].

Moreover, improved mental health outcomes were linked to digital SP use. Participants in our study believed that digital tools could reduce loneliness and promote wellbeing through better engagement and follow-up. This supports findings from evaluations of the NHS Digital Participation programme, where users reported reduced social isolation, increased confidence, and more efficient use of health services [30]. Similarly, studies have found that older adults who use the internet report higher life satisfaction and lower rates of depression [31].

Despite these benefits, participants also expressed significant concern about the risks of digital exclusion. A recurring theme was the unequal access to digital infrastructure, including internet connectivity, devices, and skills. This concern is well-documented in existing literature. The World Health Organization has cautioned that digital health technologies, while promising, risk worsening health inequalities if barriers to access are not addressed [30, 32]. Similar to our findings, other studies have shown that people with lower socioeconomic status, limited education, or poorer health are more likely to be digitally excluded [33].

Digital literacy emerged as a critical enabler for the success of digital SP. Stakeholders repeatedly mentioned the need for training and capacity-building for both service providers and users. This supports broader public health research showing that digital skills are essential for meaningful engagement with health technologies [34]. In the absence of targeted digital literacy programmes, there is a risk that digital SP tools could become underutilised or misunderstood, further excluding already marginalised populations [35].

Financial and infrastructural limitations were additional challenges raised by our participants. Some believed that long-term savings and improved efficiency would justify digital investment, while others warned of the high initial costs related to software, training, and systems maintenance [36]. These differing views are reflected in wider debates on digital healthcare implementation, with researchers like Sharman et al. arguing that without sustained funding and political will, digital SP programmes may struggle to scale [37].

Participants also identified fragmentation across digital systems as a major obstacle. Lack of interoperability between NHS systems, local authorities, and voluntary sector providers was seen as a key challenge that could hinder timely and coordinated care. This is consistent with evaluations showing that fewer than half of social care providers in the UK have access to shared digital records, limiting effective data flow [38, 39]. The need for shared standards, interoperability frameworks, and secure, centralised platforms is increasingly evident and was strongly supported by participants in our study [40].

Concerns about cybersecurity, misinformation, and data privacy were also raised. Participants warned that without robust safeguards and user education, these issues could undermine public trust in digital SP. Literature in the field has shown that concerns about online security and poor-quality health content can deter users, particularly older adults and those unfamiliar with digital platforms [41]. Stakeholders called for clear data governance protocols, training on cyber-security, and the development of validated health information standards to improve content reliability and build trust [42].

### Strengths of the study

One key strength of this study is its multi-stakeholder approach. By capturing the views of NHS service commissioners, link workers, and community providers, we were able to generate a comprehensive understanding of the opportunities and barriers associated with digital SP. The use of structured focus group discussions allowed for in-depth exploration of each theme, supported by diverse examples and real-world insights. Our thematic framework was informed by participant-led discussions, enhancing its validity and relevance to practice.

### Study limitations

This study also has limitations. First, while provider and system-level perspectives were captured, we did not include the views of service users—the end beneficiaries of digital SP. Future studies should directly involve patients and the public to understand their experiences, preferences, and digital needs. Second, our sample was geographically restricted to Northwest England and lacked demographic diversity. Broader research across more regions and with a wider participant demographic would help ensure generalisability. Lastly, while our findings provide a qualitative understanding of digital SP, further mixed-methods research including quantitative analysis is needed to evaluate long-term outcomes and cost-effectiveness.

## Conclusion

Our findings support the growing body of evidence that digital SP can enhance accessibility, coordination, efficiency, and user empowerment in health and social care. However, achieving its full potential requires substantial investment in digital infrastructure, literacy programmes, and system integration. Importantly, digital SP must be developed with an equity lens to ensure that vulnerable groups are not excluded. Stakeholders strongly emphasised the need for inclusive design, user training, reliable funding, and standardised data systems. As digital health becomes more embedded in care delivery, these considerations will be crucial to ensuring person-centred, equitable, and sustainable implementation of social prescribing in a digital age.

### Implications for future research

Future studies should evaluate the impact and effectiveness of digital SP through longitudinal designs. This would provide robust evidence on its long-term benefits and limitations. Research should also explore how digital SP apps could be co-developed with end users, ensuring their needs and preferences shape the design and functionality of these platforms.

### A reflexivity statement

*“As a research team with a background in qualitative studies*,* and psychology we approached this study with an understanding of the importance of social prescribing as an approach to take a non-clinical approach toward health. Aware of this*,* we maintained a reflexive journal throughout the research process to critically examine our assumptions and their potential impact on data interpretation. Engaging in regular discussions with colleagues and key stakeholders provided additional perspectives*,* helping to identify and address any biases that may have arisen.”*

## Data Availability

All data generated or analysed during this study are included in this published article.
